# The Effect of Botulinum Neurotoxin Serotype a Heavy Chain on the Growth Related Proteins and Neurite Outgrowth after Spinal Cord Injury in Rats

**DOI:** 10.3390/toxins10020066

**Published:** 2018-02-02

**Authors:** Ya-Fang Wang, Fu Liu, Jing Lan, Juan Bai, Xia-Qing Li

**Affiliations:** Department of Pathophysiology, Shanxi Medical University, Taiyuan 030001, China; 041510570@b.sxmu.edu.cn (Y.-F.W.); 031513156@b.edu.cn (F.L.); 041410136@b.sxmu.edu.cn (J.L.); 041510569@sxmu.edu.cn (J.B.)

**Keywords:** botulinum neurotoxins, botulinum neurotoxin serotype A, heavy chain, botulinum neurotoxin serotype a heavy chain (BoNT/A HC), spinal cord injury (SCI), nerve regeneration, growth associated protein 43 (GAP-43), superior cervical ganglion 10 (SCG10), neuronal processes, neural regeneration

## Abstract

(1) Background: The botulinum toxin A (BoNT-A) heavy chain (HC) can stimulate the growth of primary motor neurites. (2) Methods: A recombinant BoNT/A HC was injected locally plus interval intrathecal catheter of BoNT/A HC to rats with ipsilateral semi-dissociated lumbar spinal cord injuries (SCIs). First, 2D gel with a silver nitrate stain was applied to detect the general pattern of protein expression. Growth associated protein 43 (GAP-43) and superior cervical ganglion 10 (SCG10) were chosen to represent the altered proteins, based on their molecular weight and pI, and were used to further detect their expression. Meanwhile, the neuronal processes were measured. The measurements of thermal hyperalgesia and grasp power at the ipsilateral hindlimb were used to evaluate spinal sensory and motor function, respectively. (3) Results: The local injection of BoNT/A HC followed by its intrathecal catheter intervally altered the spinal protein expression pattern after an SCI; protein expression was similar to normal levels or displayed a remarkable increase. The changes in the expression and distribution of phosphorylated growth associated protein 43(p-GAP 43) and superior cervical ganglion 10 (SCG 10) indicated that the administration of BoNT/A HC to the SCI significantly amplified the expression of p-GAP43 and SCG10 (*p* < 0.05). Meanwhile, the positive immunofluorescent staining for both p-GAP43 and SCG10 was mainly present near the rostral aspect of the injury, both in the cytoplasm and the neuronal processes. Moreover, the outgrowth of neurites was stimulated by the BoNT/A HC treatment; this was evident from the increase in neurite length, number of branches and the percentage of cells with neuronal processes. The results from the spinal function tests suggested that the BoNT/A HC did not affect sensation, but had a large role in improving the ipsilateral hindlimb grasp power (*p* < 0.05). (4) Conclusions: The local injection with the intermittent intrathecal administration of BoNT/A heavy chain to rats with SCI increased the local expression of GAP-43 and SCG 10, which might be affiliated with the regeneration of neuronal processes surrounding the injury, and might also be favorable to the relief of spinal motor dysfunction.

## 1. Introduction

A spinal cord injury (SCI) is a devastating condition characterized by a sudden loss of spinal functions distal to the level of trauma, including sensory, motor, and autonomic function. These effects are due to various traumatic or non-traumatic factors. The regenerative capacity of the injured spinal cord is extremely limited in adult mammals [1,2]. Patients suffer various disabilities which greatly impact quality of life [3]. The deficiency of spinal regenerative ability after an SCI has been attributed to various alterations in the local microenvironment, including: (1) the accumulation of many inhibitory factors by the degeneration of neurons, axon, and myelin; and (2) the lack of a sufficient number of upregulated or activated neuritogenic-associated genes and proteins [4,5,6,7,8]. At present, although there are many attempts for the treatment of the SCI, such as IL-6, stem cell transplantation [9,10], etc. few are actually available for clinical practice. Therefore, endeavors towards new strategies to revitalize neuritogenic ability following spinal injuries are being continuously made.

Botulinum toxin A (BoNT/A) is one of seven botulinum toxins (categorized based on serologic features) and is one of the most potent toxins known [11,12,13,14]. The toxicity of BoNT/A is due to its selective binding to the membranous receptor located on the presynaptic membrane of the motor endplate. This leads to the cleavage of the synaptosomal-associated protein 25 kDa (or SNAP-25) which blocks the release of the neurotransmitter acetylcholine contained in the vesicles [15,16]. This results in the paralysis of skeletal muscle. Patients die of severe respiratory failure due to a lack of respiratory muscle contractions [17]. Besides the aforementioned mechanisms of BoNT/A, it has been verified that it can also inhibit or block the release of other components in synaptic vesicles of different neurons, such as the calcitonin gene related peptide (CGRP) and substance P in sensory neurons [18,19,20,21,22]. Because of the actions of BoNT/A on neurotransmitters, it has been used as therapy to relieve hyper-reactive muscle dysfunctions (e.g., torticollis and hemifacial spasm), various types of chronic pain, hyperhidrosis, etc. [23,24].

Structurally, BoNT/A consists of two polypeptide chains linked by a disulfide bond. The longer chain (MW = 100 kDa) is called a heavy chain (HC) of BoNT-A; it is mainly responsible for binding to target cells and mediating the endocytosis of the toxin. The other chain with a smaller molecular weight is a light chain (LC, MW = 50 kDa). The LC is a zinc-dependent proteolytic enzyme which contributes to the cleavage of SNAP-25 at the neuromuscular junction [15,25,26]. It had been observed by some investigators that new axonal branches formed around skeletal muscles that had been paralyzed by BoNT/A for three to four months [27,28,29]. Meanwhile, the observations in vitro showed that BoNT/A was able to promote neuritogenesis in mouse motor neuron cultures [30]. The observation of neurite sprouting associated with BoNT/A in vivo or in vitro could not simply be attributed to the toxic role of BoNT-A. Moreover, the observations form Coffield’s lab also showed that the isolated binding domain heavy chain (BoNT/A HC) has the same role as BoNT/A [30]. The signal pathways relating to the binding of the heavy chain and the receptor might also be a reasonable alternative explanation.

In our previous studies, the increased phosphorylation of Akt and ERK1/2 (growth- and survival-related intracellular signal proteins) was paralleled with neuritogenesis when adding BoNT/A heavy chain into neuro-2a cell cultures (data published in Chinese). In that study, an obvious increase in phosphorylated-ERK 1/2 was seen between one to five hours after adding 1 nmol/L of BoNT/A HC (*p* < 0.05), and an increased protein level of phosphorylated-Akt was observed mainly at 15 and 60 min (*p* < 0.05). Based on the results, it was concluded that the BoNT-A heavy chain stimulates neuritogenesis. The neuritogenic mechanism of it on Neuro-2a cells might be due to the activation of ERK1/2 and Akt phosphorylation. Meanwhile, it is reasonably presumed that BoNT/A heavy chain exerted its biological roles by modulating many intracellular signals and proteins expression, some of which may be involved in neural growth and regeneration; others are possibly related to growth inhibition. Among of these proteins, growth-associated protein-43 (GAP-43) and superior cervical ganglion 10 (SCG 10) have already been evidenced to be closely related to the axonal growth and neural regeneration [31,32,33]. Therefore, commercial recombinant BoNT/A HC was applied in this study to explore the effect of BoNT/A HC on the expression of some selective growth-associated proteins, as well as its role in neurite outgrowth in rat lumbar SCI model in vivo. Meanwhile, the motor and sensory functions of the ipsilateral hindlimb were evaluated after BoNT/A HC treatment as a therapeutic regent.

## 2. Results

### 2.1. Unilateral Lumbar SCI Model and Spinal Function Evaluation

**Figure 1:**
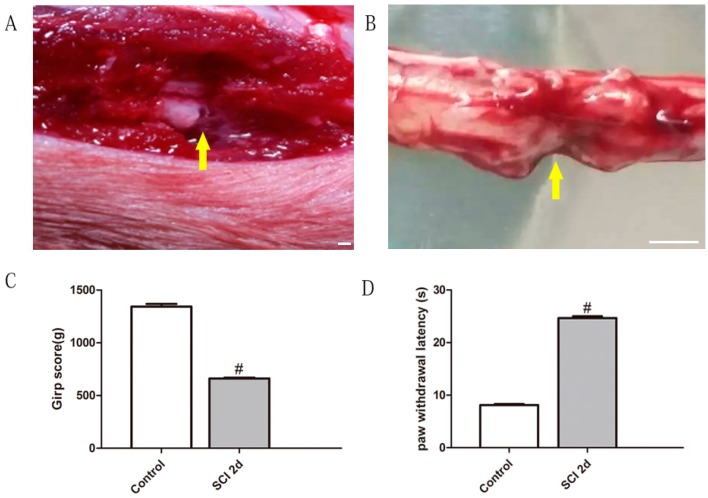
An observation of lumbar spinal cord injury (SCI) and spinal function testing. (**A**) The SCI was made by the tip of a pair of fine forceps. A clear unilateral defection was seen in the field (arrow explanation). (**B**) The lesion was located in the lumbar area when the whole spinal cord was exposed (both two arrows point to the same damaged area). (**C**,**D**) There was an obvious ipsilateral drop in the grip score and a prolonged hind-paw withdrawal latency greater than 25 s (^#^ *p* < 0.05 versus the control group; bar = 5 mm).

Observations of the SCI two days after surgery showed that it was located within the left lumbar area (Figure 1A,B). The control values for the grasp power and thermal hyperalgesia were obtained one day prior to the SCI. Two days after the SCI, both motor and sensory function appeared remarkably impaired (Figure 1C,D), i.e., the value of grasp power on grip test appeared dropped, which indicated the dysfunction of nervous control on skeletal muscle in hindlimbs, which might be related to the lesion of the corticospinal tract at lumbar spinal level, and also contributed in the appearance of the draging of the ipsilateral hindlimb.

### 2.2. The Alteration of Local Protein Expression after SCI and the Effect of BoNT/A HC on It

**Figure 2:**
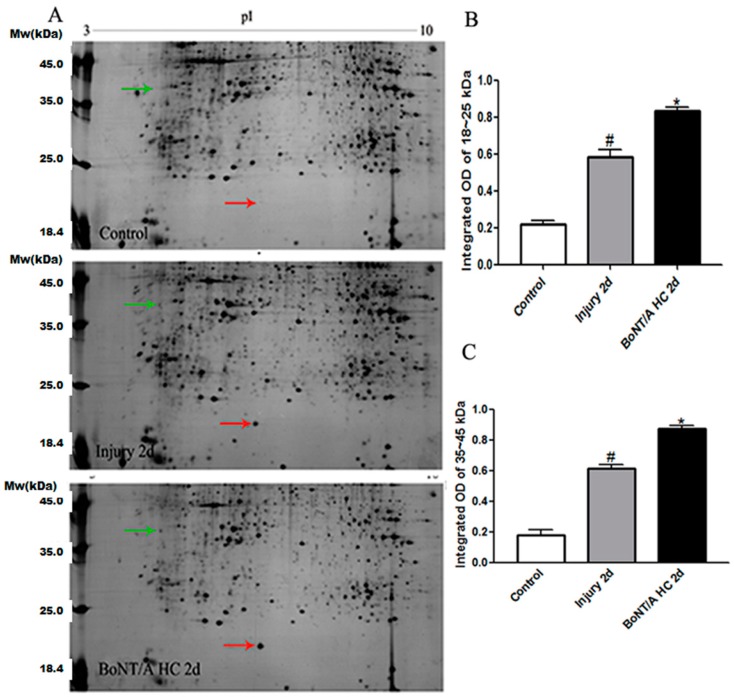
The two-dimensional gel with silver nitrate staining. (**A**) The images show that the protein dots change to molecular weights of approximately 18–45 kDa. Red and green arrows point to the dots with obvious alterative expression in the different groups. (**B**) The integrated OD value for protein expression (*n* = 4/each group) appeared at approximately 18–25 kDa. (**C**) The integrated OD value for protein expression appeared at approximately 35–45 kDa. Results were statistically significant (^#^ *p* < 0.05, vs. control group; * *p* < 0.05, vs. both control & injury groups).

Two-dimensional electrophoresis with silver nitrate staining showed that the general expression of protein in SCIs was altered compared to that of the control group. The major changes noted after injury were that some protein dots were lighter in color or smaller in size, while others appeared simultaneously denser or increased. Furthermore, the proteins with either increased or decreased expression appeared as a single dot or a dot group. After administration of the BoNT/A HC, the alterative expression of proteins demonstrated some interesting phenomena: some of the protein dots with increased expression tended to decrease towards the levels seen in the control group, whereas some dots with increased expression either further increased remarkably or went down in expression. It was noted that the protein dots that were 35–45 kDa (isoelectric point of 4 to 5) and 18–25 kDa in molecular weight (isoelectric point 7) had an interesting appearance: their expression was increased after the SCI, and then this increase became much more significant after treatment with BoNT/A HC (Figure 2).

### 2.3. Effects of BoNT/A Heavy Chain on the Expression of Selective Growth-Associated Proteins

**Figure 3:**
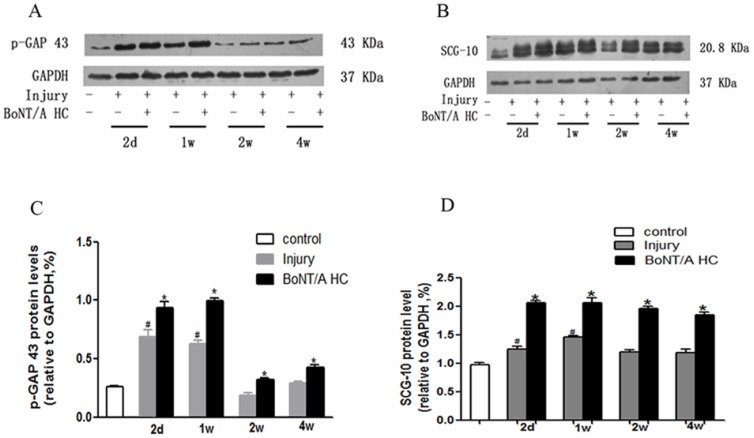
The effect of BoNT-A heavy chain (HC) on the expression of p-GAP43 and SCG10. The Western blot bands for both: p-GAP43 (**A**); and SCG10 (**B**) at different periods (two days, one week, two weeks, and four weeks). The semi-quantification analysis (4 sample/each group) of: p-GAP43 (**C**); and SCG10 (**D**). Results were statistically significant (^#^ *p* < 0.05, vs. the control group; * *p* < 0.05, vs. both the control and the injury groups).

According to the profile of the 2D gel from different group, two dots from the molecular weight (MW) between 35 and 45 kDa, Isoelectric point (pI) around 4–5 and the MW between 18 and 25 kDa, pI around 7 were selected as the aimed proteins. Based mainly on the molecular weight of some growth-associated proteins, GAP43 (MW = 43 kDa) and SCG10 (MW = 20.8 kDa) were selected as target proteins for the BoNT/A HC treatment intervention. Both SDS-PAGE and a Western blot showed that the expression of p-GAP43 and SCG10 were increased two days after the SCI while using GAPDH as a loading control. However, the increase declined as time post-injury increased, even dropping below the level of the control group two weeks after the injury. With the BoNT/A HC injection, there was a much greater increase of the expressions of the two proteins. The increase was significant compared with SCI-only samples during the corresponding period (*p* < 0.05). In comparison, SCG10 had a more increased expression than p-GAP43 after administration of BoNT-A HC; this was based on the semi-quantification of both protein bands on the Western blot. The expression level of SCG10 was almost two times greater than that of control groups and 75% greater than SCI-only groups at four weeks post-injury. Meanwhile, the expression of p-GAP43 was also increased after BoNT/A heavy chain treatment, but the change was not as dramatic as in SCG10 (Figure 3).

### 2.4. The Distribution of Positive GAP43 and SCG10 Immunofluorescence at the Injured Area after Administration of the BoNT/A HC

**Figure 4:**
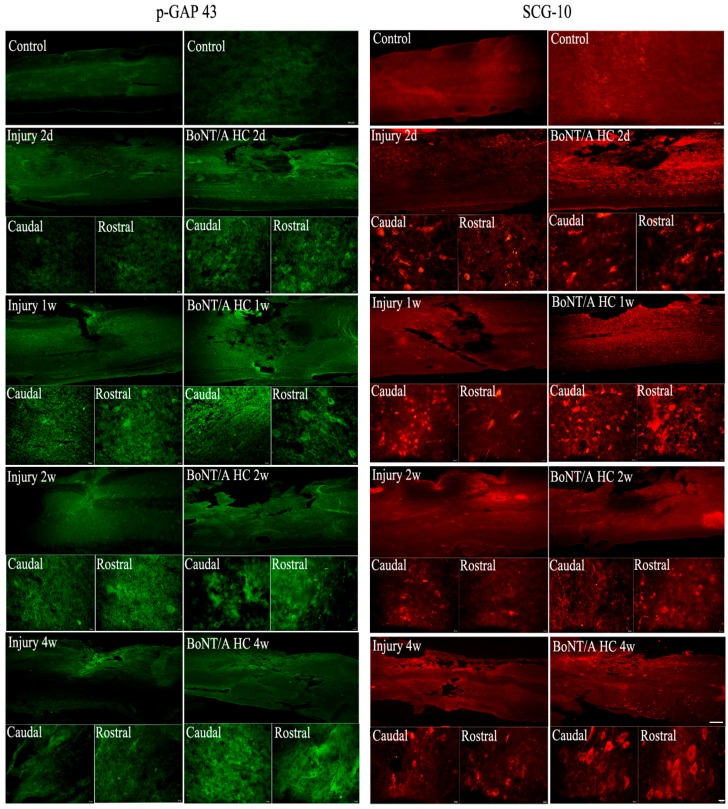
A comparison of the immunofluorescence of GAP43 (Green) and SCG10 (Red) at the spinal injury site. The scale bars were set at 500 μm (on the long rectangular image at right bottom; and 20 μm (short rectangular image at right bottom; lower sections are the magnified images from rostral and caudal sites of the corresponding upper one. Each group consists of one long rectangular and two short rectangular images), respectively.

Results showed that in a normal spinal cord both GAP43 and SCG10 are evident in only a few cells with weak positive expression. Under the microscope, the positive immunofluorescence of GAP43 and SCG10 at the injury area was consistent with the results of the Western blot. The distributive features of the two proteins appeared very similar. The positive GAP43 and SCG10 expression was mainly within the cell body. These cells were very evident during the immunofluorescence of both proteins; they were found in the caudal and rostral parts of the injury area two to seven days after the injury, with or without BoNT/A HC administration. With BoNT/A HC treatment, however, the immunopositivity of both proteins appeared more intense than the SCI-only groups after two weeks post-injury. Furthermore, with BoNT/A heavy chain treatment, the positive staining was observed not only within the cytoplasm but also in the cellular processes; this made the processes easily identifiable. Comparably, the neuronal processes were much more obvious in the immunofluorescence of SCG10 than of GAP43 (Figure 4). Both Western blot and immunofluorescence results imply that SCG10 might be an important growth-associated protein affected by the BoNT/A HC. The positive SCG10 neuronal processes were used as the target for the measurement of neurite length and number after in vivo administration of BoNT/A HC.

### 2.5. The Measurement of SCG 10 Positive Neuronal Processes

**Figure 5:**
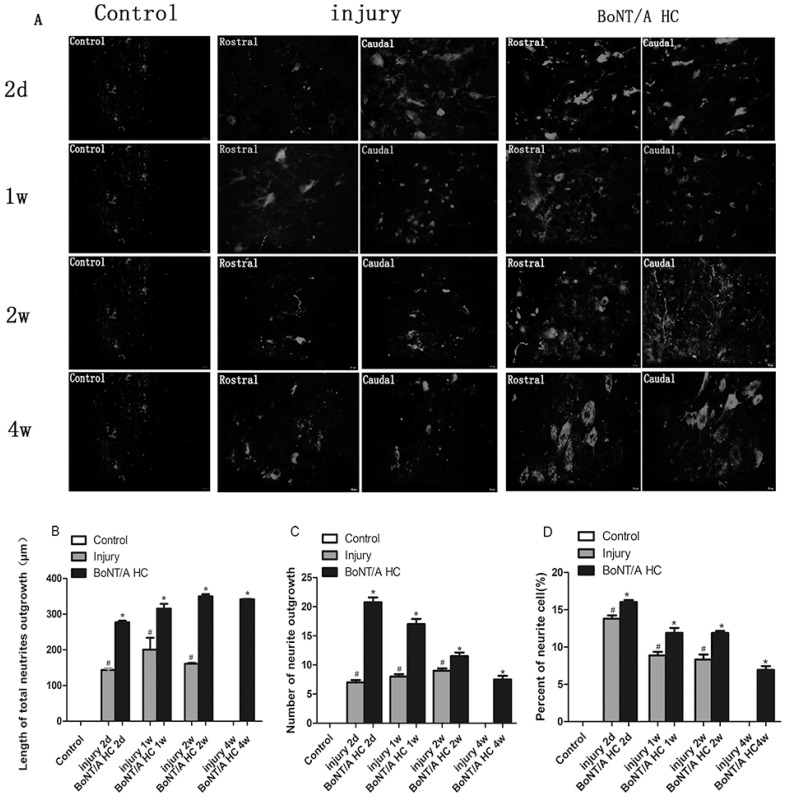
The effects of BoNT/A HC on the neurite outgrowth. (**A**) The visualization of neuronal processes and their variation in different groups using SCG10 immunofluorescence. A quantitative comparison of the: total length (**B**); number (**C**); and the percentage of neurons with visualized processes in all SCG10 positive cells (**D**). Results were statistically significant (^#^ *p* < 0.05, vs. control group; * *p* < 0.05, vs. both control and injury groups); Bar = 20 μm.

Based on immunofluorescence, the length and number of neuronal processes with SCG10 positivity at the caudal and rostral areas of injury were imaged and measured using ImageJ software (a free software from https://imagej.nih.gov/ij/). (The number of SCG 10 positive neurons was various from group to group, but the variation between group and group, especially SCI and BoNT/A HC treatment group, was not significant (*p* > 0.05). The calculation appeared as 35–45 (in controls), 55–65 (in SCI 2 d), 80–90 (SCI 1 w), 90–100 (SCI 2 w), 80–90 (SCI 4 w), 70–80 (with BoNT/A HC 2 d), 100–105 (with BoNT/A HC 1 w), 100–105 (with BoNT/A HC 2 w) and 90–100 (with BoNT/A HC 4 w). SCG10 positive processes were scattered, short, and not easily identified one week after the SCI; this was true even regarding the positive cells that were clearly observed in that time. With lasting post-injury time (four weeks), these neuronal processes eventually disappeared in the SCI-only group. In contrast, the number of neuronal processes significantly increased when BoNT/A HC was given. The total length of positive SCG10 neurites reached 400 μm in four weeks, and the process branches were observed to increase even two weeks after injury. The increase in neuronal number was almost three times greater than control and SCI-only groups (*p* < 0.05) (Figure 5). Additionally, the percentage of positive SCG10 cells with identified neurites was higher than in SCI-only groups at the corresponding time (*p* < 0.05) (Figure 5). These results indicate that BoNT/A HC was able to stimulate the re-growth of neuronal processes after an SCI, as previously seen in vitro studies.

### 2.6. The Amelioration of Motor and Sensory Function by BoNT/A HC Treatment Based on SCI

**Figure 6:**
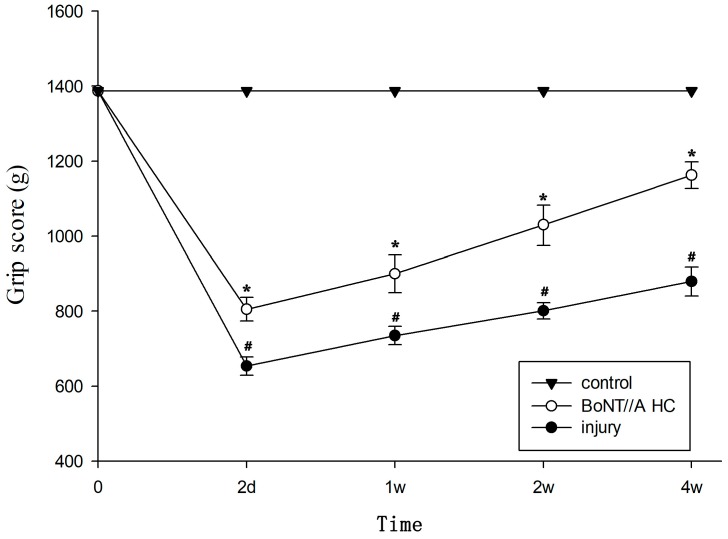
The motor function changes in the ipsilateral hindlimb after BoNT/A HC treatment. Results were statistically significant (^#^ *p* < 0.05, vs. control group; * *p* < 0.05, vs. injury group).

The ipsilateral hindlimb motor function was evaluated using grip testing at two days, one week, two weeks, and four weeks post-SCI. Results showed that there was a significant drop in grip scores, which indicated that there was some damaged motor function of the ipsilateral hindlimb after the SCI. However, rats administered with BoNT/A HC treatment showed relatively higher scores than those in the SCI-only group (*p* < 0.05), though their scores were not as high as those of the control group (Figure 6). 

The test for thermal hyperalgesia was used to assess sensory function. The results indicated that the paw withdrawal latency (PWTL) was prolonged after an SCI, with or without the application of BoNT/A HC, and there was no significant difference between the SCI groups and the BoNT/A HC treatment groups (Table 1).

## 3. Discussion

The stimulatory effect of BoNT/A HC on neurite outgrowth has previously been verified in vitro. In this work, the intermittent administration of BoNT/A HC through intrathecal injection on a lumbar unilateral SCI rat model was used to determine the role of BoNT/A HC in the local expression of growth-associated proteins. It was then explored whether, in vitro, BoNT/A HC was able to promote re-growth by upregulating the expression of growth-associated proteins after an SCI. Unlike the peripheral nervous system (PNS), the central nervous system (CNS) is deficient in its ability to regenerate after injury [34,35]. Based on previous research, the CNS has difficulty regenerating due to the abundance of myelin inhibitory factors and the shortage of macrophages [6]. Another possible explanation is the limited or arrested initiation of intrinsic neuron growth programs in the injured CNS [7]. Because the spine is part of the CNS, most patients suffer from severe spinal dysfunction and lifelong disability due to various pathogeneses when SCIs develop. Therefore, one of the principal foci of regenerative medicine after an SCI is to explore strategies and methods which can influence the improvement of the spinal microenvironment (e.g., the exclusion of myelin inhibitory factors) or revive the intrinsic regenerative ability of neuron itself [36].

The human recombinant BoNT/A HC used in this study is a non-toxic peptide, unlike the original potential holotoxin. The results evident after treatment with BoNT/A HC can be explained by its ability to bind to membranes and the activation of related intracellular signals upon the attachment of the heavy chain to the receptor. A previous study from the same research team has demonstrated that the BoNT/A HC had a stimulatory role in promoting neurite outgrowth in Neuro-2a cell cultures. It is known that the basis for neurogenesis and neuritogenesis is the synthesis of proteins; even the myelin inhibitory factors that accumulate after SCI can be regarded as a secondary phospholipoprotein. Therefore, it can be assumed that there is a significant modification in protein expression after an SCI, and the administration of the BoNT/A HC following an SCI should have an input in protein expression. Some definite results were obtained from this study. First, the application of BoNT/A HC reversed the alterations caused by the SCI, such as the increased expression of some proteins when the SCI developed. When the BoNT/A HC was given at the same time as the injury established, the increase dropped towards the levels of the controls, which is consistent with our previous study (published in Chinese). Briefly, the main difference in the previous study are as foolows: (1) the SCI model was established with a modified needle inserted into the lumbar spinal cord at the same anatomical location as this study; and (2) different doses of BoNT/A heavy chain (2 μg, 4 μg, 6 μg and 8 μg) was injected one-time into the spinal cord cavity while the injury made. The second result observed in this study is that BoNT/A HC treatment magnified the expression of some proteins evident from the SCI, for example, the expression of GAP43 and SCG10 were obviously increased after BoNT/A HC treatment. In this study, the selection of GAP43 and SCG10 as markers (to detect the efficiency of BoNT/A HC on stimulating protein regeneration) is based on the changes of protein molecular weight on the two-dimensional gel and the Western blot. In fact, the levels of both GAP43 and SCG10 displayed a certain elevation shortly after the SCI (two days post-injury), but these declined as the post-injury period progressed. However, when the SCI model was treated with BoNT/A HC, both proteins exhibited a continuous increased expression until the end of the experiment.

The increased expression of GAP43 after injury has been considered by most researchers to be actively involved in axonal regeneration [37,38,39,40,41]. GAP43 is mainly involved in the sprouting and regeneration of mature axons in their phosphorylated form. Its expression was upregulated following nerve injury. SCG10, also known as stathmin-like 2 (STMN2) protein, is another regenerative protein [42]. It is mainly involved in axonal microtubule dynamics and protein transport [43,44,45]. The initial increase in GAP43 and SCG10 expression after the SCI, without the application of BoNT/A HC, can be explained as a limited regenerative response after spinal cord injuries, however the response ceased when the injury continued to exist. The mechanism might be related to the intervention of myelin inhibitory factors binding to their specific receptors. The continuous enhancement of GAP43 and SCG10 expression when BoNT/A HC is applied is evidence of the recombinant peptide’s involvement in promoting nerve regeneration after injury. The study also gave an outline of the role BoNT/A HC plays in stimulating the in vivo sprouting of neuronal processes. When BoNT/A HC treatment was administered during the SCI’s development, the length and the number of neuronal process around injury site (in both its rostral and caudal parts) increased compared to measurements in the SCI-only group. The percentage of neurons with processes was also greater than that in the SCI-only group. Additionally, it was found that the spinal motor function at the ipsilateral hindlimb was improved due to the efficiency of BoNT/A HC in the upregulation of growth-associated proteins and in the promotion of neuronal process re-growth. The ineffectiveness of BoNT/A HC on improving spinal sensory function might be attributed to the difference in the expression of the heavy chain binding receptor in sensory neurons. Besides, direct damage of the sensory neurons in injury location might contribute to the difference of sensory and motor after SCI with or without BoNT/A HC treatment. Therefore, more detailed information about these should be explored after all in the future.

## 4. Conclusions

The local injection with intermittent intrathecal administration of BoNT/A heavy chain to rats with SCI increased the local expression of GAP-43 and SCG 10, which might be affiliated with the regeneration of neuronal processes surrounding the injury, and might also be favorable to the relief of spinal motor dysfunction. The exact role and mechanism in vivo of BoNT/A heavy after nervous injury need to be verified in the future.

## 5. Materials and Methods

### 5.1. Establishing the Rat Spinal Cord Hemi-Section Injury Model

All experimental procedures were done in accordance with the National Institute of Health’s Guide for the Care and Use of Laboratory Animals, and were approved by the Ethics Committee of Animal Research at Shanxi Medical University (IACUC2017-001) on 20 January 2017. Every effort was made to minimize animal suffering and the number of animals sacrificed.

Sprague-Dawley rats (6–7 weeks old, and weighing between 200 and 220 g) were provided by Beijing Haidian Experimental Animal Farm (No. SCXK (Beijing) 2014-0013). As there is no difference regarding spinal cord injury study, male rats were used in this research. The rats were deeply anaesthetized with 1% pentobarbital sodium (at a dose of 4 mL/kg body weight) and given a laminectomy at vertebral level T_9_/_10_ to expose the spinal cord. Brieﬂy, the T_9_ and T_10_ vertebrae were first orientated using the iliac crest as an anatomical landmark. The lumbar spinal cord was exposed by removing the T9 vertebra dorsally. A unilateral (left) lumbar spinal injury was achieved by nipping against the left side of the dorsal median vein with the tip of a pair of fine forceps. Left hindlimb paralysis was regarded as the sign of success in achieving a unilateral SCI. Meanwhile, the loss of motor function (based on hindlimb grasp power measurement) and the abnormality of sensory function (based on an assay of thermal hyperalgesia) after surgery were used to evaluate the severity of spinal cord function impact. The animals were placed in a temperature-controlled (24 °C ± 1 °C) chamber under a 12 h light/dark cycle. Standard amounts of food and tap water were given daily.

### 5.2. BoNT/A Heavy Chain Administration and Groups

Recombinant BoNT-A HC was purchased from List Biological Laboratories Inc. (Campbell, CA, USA).

Intermittent administration of BoNT/A HC applied in the study was done via two routes: (1) local application of the BoNT/A HC directly onto injury site (4 μg/μL in 16 μL saline); and (2) administration of BoNT/A HC via a lumbar intrathecal catheter [46]. After establishing the rat model, 2 μg/μL of BoNT/A HC were administered every week to the SCI in the BoNT/A HC treatment groups. Animals were divided into the following groups, with six rats in each group: (1) the control (or pseudo surgery) group for which the skin incisions were made, the laminae were cut, and the spinal cords were exposed but not injured; (2) the SCI-only group for which unilateral (left side) lumbar spinal cord injuries were made (to prevent the vehicle intervention on BoNT/A heavy chain, the same volume of sterile saline was applied in SCI-only animal with same method and period); and (3) the SCI with BoNT/A HC treatment, in which BoNT/A HC was administered every week, and the injury was made. This group was further divided into four groups (with six rats each) based on different periods post-injury, i.e., two days, one week, two weeks and four weeks. The groups of animals are summarized in Table 2.

### 5.3. Two-Dimensional Gel Electrophoresis

The spinal cords were fully exposed when the experiment’s period was completed. Then, the segment of the cord containing the spinal lesion center and the rostral and caudal area (6 mm in total) was collected. Spinal protein was extracted using a pre-cooled lysis buffer (urea: 21 g, thiourea: 7.9 g, DTE: 0.5 g, Tris: 0.5 g, in 50 mL of DD H_2_O). The protein concentration was determined using the Bradford protein assay.

For the two-dimensional gel, two 150 mg protein samples from each group were loaded onto IPG strips (NL) for first-dimension isoelectric focusing (IEF). The IPG strips were then washed and equilibrated in a 2D equilibration buffer, and then they underwent a 12.5% sodium dodecyl sulfate polyacrylamide gel vertical electrophoresis. After that, the gel was stained with 0.1% silver nitrate. The gel was imaged and analyzed with the Bio-Rad gel imaging system. Some interesting protein dots from different group were chosen and compared upon their changes on size and density at the same level of molecular weight and the isoelectric point (pI). For statistical analysis, two gels were prepared. The selective dots were circled and assessed by the integrated optional density (IOD) using ImageJ software in a double-blinded way and two people who were not related to the study were asked to do the assessment. In this way, four values four each dot were obtained and analyzed.

### 5.4. SDS-PAGE and Western Blot

Twenty micrograms of protein homogenates were subjected to 15% sodium dodecyl sulfate polyacrylamide gel electrophoresis. Subsequently, the proteins were transferred onto polyvinylidene difluoride (PVDF) membranes for Western blotting. The PVDF membranes were blocked with 5% non-fat milk powder in a TBST wash buffer (Tris buffered saline containing 1% Tween-20) for 1 h at room temperature. The selective primary antibodies were applied onto the membrane and incubated at 4 °C overnight. These antibodies were the following: rabbit anti-phosphorylated GAP43 antibody (1:3000, Thermo Fisher, Waltham, MA, USA); rabbit anti-SCG10 polyclonal antibody (1:1000, Thermo Fisher, Waltham, MA, USA); and rabbit anti-GAPDH polyclonal antibody (1:5000, Bioword, Nanjing, China). The following day, the membranes were incubated with HRP-conjugated secondary antibodies (goat anti-rabbit IgG, 1:1000, Bioword, Nanjing, China) for 1 h at room temperature. The membranes were then immersed into the EasySee Western blot kit (DW101, TRAN, Beijing, China; A solution: B solution = 1; 1 plus 2 μL C solution) and made to undergo the reaction for 3 min in dark. X-ray film which corresponded to the membrane was developed in a darkroom. The protein bands were imaged and analyzed for the variation in integrated optional density (IOD), while GAPDH bands as a loading control, with Bio-Rad gel imaging system.

### 5.5. Immunofluorescence Staining

At the set time point, rats from each group were anesthetized lethally with 1% pentobarbital sodium. They then underwent a 30 min transcardial perfusion with ice-cold 4% paraformaldehyde for fixation purposes. A 1 cm segment of spinal cord containing the lesion center was dissected out. The samples were post-fixed in the same fixatives for another hour and subsequently immersed in a 30% sucrose solution at 4 °C overnight. After being embedded onto an OCT compound, a section of spinal cord 16 μm in thickness across the lesion site was cut dorsal-coronary-longitudinally by cryostat (1950-Cryostat, Leca, Nussloch, Germany). 

The sections were penetrated with 0.1% Triton-X100 for 10 min, washed three times with 0.1 mol/L PBS buffer, and blocked with a blocking buffer (10% goat or donkey serum and 0.1% Triton X-100 in PBS) for 1 h. Primary antibodies (as previously listed) were incubated with the section at 4 °C overnight. The following day, the sections were incubated with Alexa Fluoro-594-labeled goat anti-rabbit IgG (1:500; Life Technologies, Shanghai, China) and Alexa Fluoro-488-labeled donkey anti-rabbit IgG (1:500; Life Technologies, Shanghai, China) in the dark for 1 h, at room temperature. The sections were then mounted with an antifade mountant with DAPI (2-(4-amidinophenyl)-1H-indole-6-carboxamidine, Life Technologies, Grand Island, NY, USA). These were then checked and photographed under a fluorescence microscope (Olympus IX71, Tokyo, Japan). 

### 5.6. Measurement of Neuronal Processes

The immunofluorescence staining of SCG 10 was applied to 6–8 sections each samples. Approximately 5–7 of images were captured of each section. The imaging area was mainly at the periphery of the spinal cord lesion. The measurements included the total length of neurites, the number of neurites, and percentage of cells with neurites in all the immunofluorescence positive cells in the image. These were taken using ImageJ software. The identification of neurites was determined and confirmed according to literature [47]. Neurites were traced from the cell body to the end of the process, and the total length was calculated for each process. Neurites were distinguished according to their soma diameter; this meant that only neurites with a length greater than the maximum diameter of the cell would be considered a real neurite. 

### 5.7. Behavioral Evaluation

Six rats in each group were used for assessment of motor and sensory function of ipsilateral hindlimb. Alteration in sensory and motor function at the ipsilateral hindlimb of rats with an SCI or an SCI with BoNT/A HC treatment were evaluated using the thermal hyperalgesia test and the grip power test, respectively. The thermal hyperalgesia test was completed using thermal stimulation of the PWTL by the paw thermal radiation stimulation tester (SERIES 8; RWD Life Science). The response time (in seconds) for the ipsilateral paw to withdraw because of the thermal stimulus was recorded. In fact, the response time represented the latency of the stimulation of the rat’s foot (accurate to 0.1 s). To avoid tissue damage caused by long time thermal stimulation, 25 s was regarded as the maximum time of PWTL. At every time point, the average value was obtained using at least three measurements. A 10 min interval was suggested between taking these measurements. The values from three or more assessments was applied to calculate the average ± SD at a certain time point.

The grip test was performed using the rats grip tester based on the manufacturer’s instructions (YLS-13A, Zhishuduobao, Baoding, China). First, wrap two fore-limbs and one counter-lateral hindlimb with tape, leaving the ipsilateral hind-limb free. Then, the rat was placed gently on the grasping force tester, the rat’s tail was grabbed and dragged backwards. The power (in grams) of the ipsilateral hind paw in holding the grip was automatically recorded by the instrument as the maximum rat grip power; this stands for its motor function. The value was the average of three measurements, with 15 min intervals between each measurement.

### 5.8. Statistical Analysis

The data were expressed as mean and standard error (SEM). The protein bands or protein spots were quantified by ImageJ software. Data were analyzed using GraphPad Prism 5.0 software and one-way ANOVA was used to analyze the significance. *p* < 0.05 was considered statistically significant.

## Figures and Tables

**Table 1:** Comparison of thermal pain thresholds at different time points in different groups.

Groups	0 d	2 d	1 w	2 w	4 w
Control	8.5 ± 0.2	8.5 ± 0.2	8.3 ± 0.1	7.8 ± 0.2	8.2 ± 0.2
Injury	8.5 ± 0.2	22.8 ± 1.0 ^#^	22.1 ± 0.9 ^#^	22.4 ± 1.0 ^#^	21.5 ± 0.9 ^#^
BoNT/A HC	8.5 ± 0.2	24.4 ± 0.3	24.5 ± 0.3	24.1 ± 0.4	22.5 ± 0.9

^#^ *p* < 0.05, vs. control group.

**Table 2:** Number and Groups of Animals.

Name of Group	Pseudo Surgery	SCI without BoNT/A HC				SCI with BoNT/A HC			
Time	0	2 d	1 wk	2 wks	4 wks	2 d	1 wk	2 wks	4 wks
No. of animals	6	6	6	6	6	6	6	6	6
